# Structure-Activity Relationships of Acyclic Selenopurine Nucleosides as Antiviral Agents

**DOI:** 10.3390/molecules22071167

**Published:** 2017-07-12

**Authors:** Pramod K. Sahu, Tamima Umme, Jinha Yu, Gyudong Kim, Shuhao Qu, Siddhi D. Naik, Lak Shin Jeong

**Affiliations:** Research Institute of Pharmaceutical Sciences, College of Pharmacy, Seoul National University, Seoul 08826, Korea; pramodksahu@gmail.com (P.K.S.); jhini19@gmail.com (T.U.); jinha.yu@nih.gov (J.Y.); gdkim0217@gmail.com (G.K.); shuhaoqus@gmail.com (S.Q.); siddhinaik40@gmail.com (S.D.N.)

**Keywords:** antiviral, acyclic selenopurine nucleoside, prodrug, anti-herpetic

## Abstract

A series of acyclic selenopurine nucleosides **3a**–**f** and **4a**–**g** were synthesized based on the bioisosteric rationale between oxygen and selenium, and then evaluated for antiviral activity. Among the compounds tested, seleno-acyclovir (**4a**) exhibited the most potent anti-herpes simplex virus (HSV)-1 (EC_50_ = 1.47 µM) and HSV-2 (EC_50_ = 6.34 µM) activities without cytotoxicity up to 100 µM, while 2,6-diaminopurine derivatives **4e**–**g** exhibited significant anti-human cytomegalovirus (HCMV) activity, which is slightly more potent than the guanine derivative **4d**, indicating that they might act as prodrugs of seleno-ganciclovir (**4d**).

## 1. Introduction

**Figure 1:**
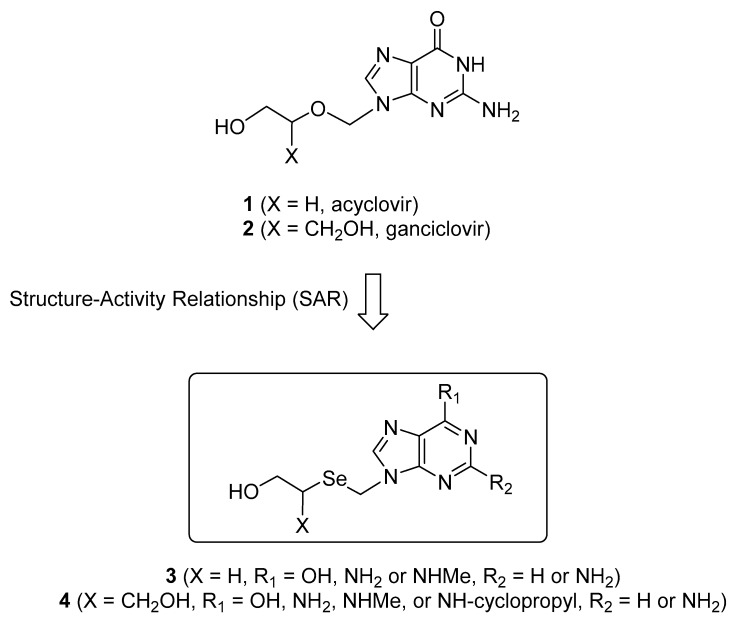
The rationale for the design of acyclic selenopurine nucleosides **3** and **4** based on the potent antiviral activity of **1** and **2**.

Modified nucleosides have continued to be fruitful resources for the development of antiviral agents [1]. Among these, acyclic nucleosides have been clinically used as drugs of choice for the treatment of herpetic viral infections such as herpes simplex virus (HSV), varicella-zoster virus (VZV), and human cytomegalovirus (HCMV) [2]. For example, acyclovir (**1**) [3,4] is widely used for the treatments of HSV- and VZV-infected patients, while ganciclovir (**2**) [5,6] is clinically used for the treatment of HCMV infections (Figure 1).

Compound **1** is intracellularly phosphorylated by viral-encoded thymidine kinase to its monophosphate, which is further phosphorylated to its triphosphate by cellular kinases. This triphosphate inhibits viral DNA polymerase reversibly by competing with the natural substrate, 2′-deoxyguanosine-5′-triphosphate (dGTP), and/or being incorporated into viral DNA chains, resulting in viral DNA chain termination [7,8]. On the other hand, compound **2** is also converted to its monophosphate by viral-encoded kinase (phosphotransferase), which is then subsequently converted into the triphosphate by cellular kinase [9]. This triphosphate inhibits HCMV DNA polymerase with a mechanism of action similar to **1** [9]. However, these drugs **1** and **2** have exhibited drawbacks such as hepatotoxicity [10], poor water solubility, and the appearance of resistant strains [11]. Although their poor water solubility has been solved by the amino acid ester prodrug approach, hepatotoxicity and resistance problems should still be solved. Thus, it has been highly desirable to develop new antiviral agents to tackle these problems.

In preliminary accounts, we synthesized the selenium analogues **3** (X = H, R_1_ = OH, R_2_ = NH_2_, seleno-acyclovir) and **4** (X = CH_2_OH, R_1_ = OH, R_2_ = NH_2_, seleno-ganciclovir) of **1** and **2** because they are in bioisosteric relationships; we also evaluated them for antiviral activity [12]. As expected, seleno-acyclovir exhibited potent anti-HSV activity, while seleno-ganciclovir exerted significant anti-HCMV activity [11]. Thus, based on these findings, it was very interesting to carry out the structure-activity relationship (SAR) study modifying the C2 and/or C6 positions of the guanine base of seleno-acyclovir and seleno-ganciclovir, which might overcome the drawbacks such as high cytotoxicity and poor water solubility caused by the guanine base. Herein, we report the full accounts of acyclic selenopurine nucleosides **3** and **4** modified at the C2 and/or C6 position as antiviral agents.

## 2. Results and Discussion

### 2.1. Chemistry

**Scheme 1:**
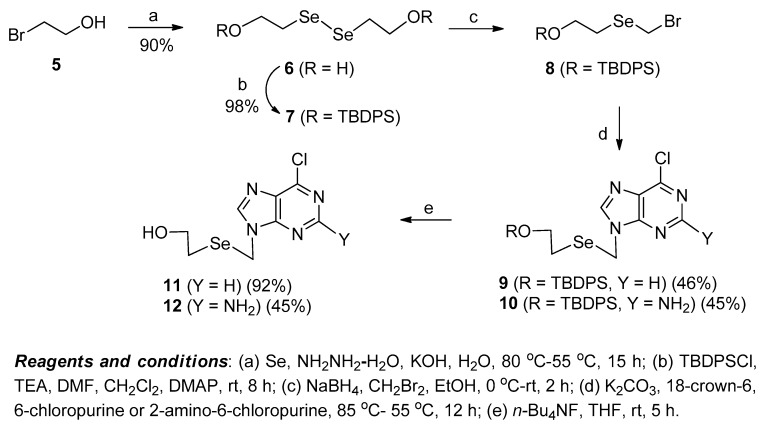
Synthesis of 6-chloro- and 2-amino-6-chloropurine derivatives **11** and **12**.

Scheme 1 illustrates the synthesis of 6-chloro- and 2-amino-6-chloropurine derivatives **11** and **12**, which serve as the versatile intermediates for the synthesis of various seleno-acyclovir analogues [11]. 2-Bromoethanol (**5**) was converted to the glycosyl donor **8** according to our previously reported procedure [12]. 2-Bromoethanol (**5**) was treated with selenium dianion prepared in situ by heating with selenium powder and hydrazine hydrate in aqueous KOH solution to afford the diselenide **6**, which was protected with the *tert*-butyldiphenylsilyl (TBDPS) group to give **7**. Treatment of **7** with NaBH_4_ followed by trapping with methylene bromide yielded the glycosyl donor **8**. Condensation of **8** with 6-chloropurine and 2-amino-6-chloropurine in the presence of K_2_CO_3_ produced the desired *N*^9^-6-chloropurine derivative **9** and *N*^9^-2-amino-6-chloropurine derivative **10**, respectively, along with concomitant formations of the corresponding N^7^-isomers in negligible amounts. The *N*^9^-isomers were confirmed by the chemical shift of the C5 signal of ^13^C-NMR. The pronounced difference in *N*^7^- and *N*^9^-isomers reside in the C5 signals of ^13^C-NMR [13]. In general, the C5 signal (~132 ppm) in the *N*^9^-isomer is shifted downfield by ~10 ppm relative to the corresponding shift in the *N*^7^-isomer [13]. The chemical shift of the C5 in **9** was 131.69 ppm, indicating that it is a N^9^-isomer. The *N*^9^-isomer **9** was further confirmed after being converted to the final **4a**. Removals of the TBDPS group in **9** and **10** with n-Bu_4_NF afforded the key intermediates **11** and **12**, respectively.

**Scheme 2:**
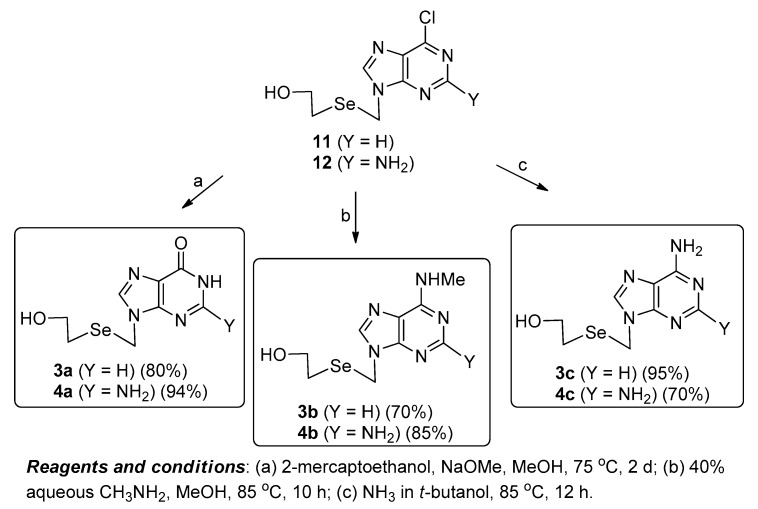
Synthesis of seleno-acyclovir analogues **3a**–**c** and **4a**–**c**.

Conversion of the key intermediates **11** and **12** into the seleno-acyclovir analogues **3a**–**c** and **4a**–**c** is shown in Scheme 2. Treatment of **11** and **12** with 2-mercaptoethanol and NaOMe in MeOH yielded seleno-inosine derivative **3a** (80%) and seleno-acyclovir **4a** [12] (94%), respectively. The *N*^9^-isomer **4a** was further confirmed by comparing the C5 signal (δ 116.73 ppm) of **4a** with that (δ 116.73 ppm) of acyclovir (**1**) [12]. Compounds **11** and **12** were also converted to the *N*^6^-methyladenine derivative **3b** and the 2-amino-*N*^6^-methyladenine derivative **4b**, respectively, by heating with 40% aqueous methylamine solution in MeOH. To overcome the low water solubility of the hypoxanthine or guanine base, **11** and **12** were transformed to the adenine derivative **3c** and 2,6-diamino derivative **4c**, respectively, by heating with *t*-butanolic ammonia at 85 °C.

**Scheme 3:**
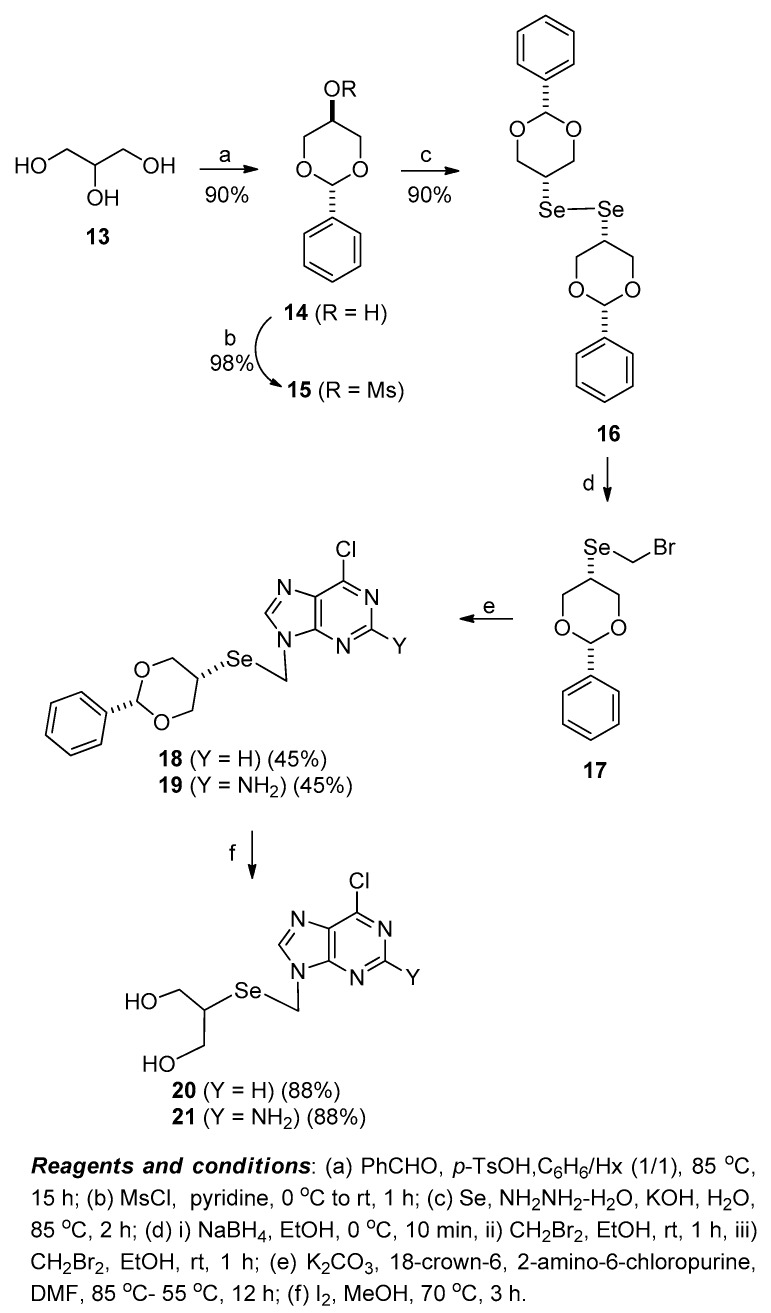
Synthesis of key intermediates 6-chloropurine derivative **20** and 2-amino-6-chloropurine derivative **21** starting from glycerol (**13**).

For the synthesis of seleno-ganciclovir analogues, the key intermediates 6-chloropurine derivative **20** and 2-amino-6-chloropurine derivative **21** were first synthesized, starting from glycerol (**13**), as depicted in Scheme 3 [12]. Glycerol (**13**) was converted to the glycosyl donor **17** according to our previously reported procedure [12]. Glycerol (**13**) was protected with the 1,3-benzylidene group by treating with benzaldehyde in the presence of *p*-TsOH to give **14**, which was treated with MsCl to give the mesylate **15**. Using similar conditions (Se, NH_2_NH_2_-H_2_O, and KOH) employed in the preparation of **6**, compound **15** was smoothly converted to the diselenide **16**. The reduction of diselenide **16** with NaBH_4_ followed by the treatment with CH_2_Br_2_ yielded the glycosyl donor **17**. Without purification, compound **17** was condensed with 6-chloropurine and 2-amino-6-chloropurine using the same procedure employed in the preparation of **9** and **10** to afford **18** and **19**, respectively. The removal of 1,3-benzylidene in **18** and **19** was achieved by treating with iodine in MeOH to give **20** and **21**, respectively.

**Scheme 4:**
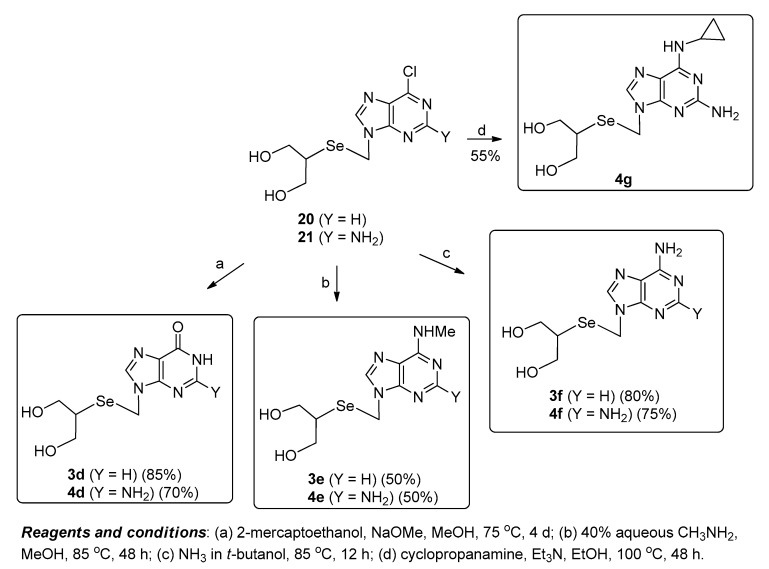
Synthesis of seleno-ganciclovir analogues **3d**–**f** and **4d**–**f**.

The key intermediates **20** and **21** were converted to the seleno-ganciclovir analogues **3d**–**f** and **4d**–**g**, as shown in Scheme 4. Treatment of **20** and **21** with 2-mercaptoethanol and NaOMe afforded the seleno-inosine derivative **3d** and the seleno-ganciclovir **4d** [12], respectively. The *N*^9^ regiochemistry of **4d** was further confirmed by comparing the C5 signal (δ 116.56 ppm) of **4d** with that (δ 116.40 ppm) of ganciclovir (**2**) [12]. The 6-chlororpurine derivative **20** was converted to the *N*^6^-methyladenine derivative **3e** and the adenine derivative **3f** by treating with methylamine and ammonia, respectively. The 2-amino-6-chloropurine derivative **21** was treated with methylamine, ammonia, and cyclopropylamine to afford the 2-amino-*N*^6^-methylaminopurine derivative **4e**, the 2,6-diaminopurine derivative **4f**, and the 2-amino-*N*^6^-cyclopropylaminopurine derivative **4g**, respectively.

### 2.2. Antiviral Activity

All the final compounds **3a**–**f** and **4a**–**g** were assayed for their antiviral activity against several herpes viruses such as HSV-1 (strain F, VR-733), HSV-2 (strain MS, VR-540), VZV (Ellen, VR-1367), and HCMV (Davis, VR-807) [12,14]. Cytotoxicity data were measured in HEL299 (CCL-137) cells as described previously [12,14]. As shown in Table 1, compound **4a** exhibited the most potent anti-HSV-1 and HSV-2 activities, whereas compound **4e** exerted the most potent antiviral activity against HCMV, although it is moderate. It is interesting to note that 2,6-disubstituted nucleosides exhibited antiviral activity, but 6-substituted nucleosides were totally inactive up to 100 µM, regardless of the substitution at the X. Compounds **4b** and **4c** also exhibited significant antiviral activities against HSV-1 and HSV-2, indicating that they might be deaminated by the cellular nucleoside deaminase to serve as prodrugs of seleno-acyclovir (**4a**), although this should be confirmed by an adenosine deaminase test. Similarly, compounds **4e**–**g**, which seem to act as prodrugs of seleno-ganciclovir (**4d**), also exhibited significant anti-HCMV activity and they were more potent than the parent compound **4d**. However, all compounds were neither active nor toxic up to 100 µM against VZV. The finding that all synthesized compounds were less potent than the reference compounds **1** and **2** might be explained by the difficulty in phosphorylation induced by steric effects of the bulky selenium atom, although it was much relieved by the freely rotatable acyclic single bond [15].

## 3. Materials and Methods

Proton (^1^H) and carbon (^13^C)-NMR spectra were recorded on a Bruker AV 400 (Bruker Corporation, Rheinstettern, Germany) (400/100 MHz) spectrometer. Chemical shifts are reported in ppm (δ) with residual solvents as the internal standard. UV spectra were recorded on a PerkinElmer Lambda25 in methanol. Melting points were determined on a Barnstead Electrothermal 9100 instrument. Mass spectra were recorded on a fast atom bombardment (FAB). All reactions involving air- or moisture-sensitive conditions were routinely carried out under an inert atmosphere of dry nitrogen. Reactions were checked by thin layer chromatography (Kieselgel 60 F254, Merck, Darmstadt, Germany). Spots were detected by viewing under UV light, and by colorizing with charring after dipping in a *p*-anisaldehyde solution. The crude compounds were purified by column chromatography on silica gel (Kieselgel 60, 230–400 mesh, Merck, Darmstadt, Germany). All solvents were purified and dried by standard techniques just before use.

### 3.1. General Procedure for Base Condensation

To a stirring solution of **7** [12] or **16** [12] (5.52 mmol) in ethanol (25 mL), NaBH_4_ (46.88 mmol) was added at 0 °C, followed by the addition of a solution of CH_2_Br_2_ (683.47 mmol) in ethanol (100 mL). The reaction mixture was stirred at room temperature for 1 h and evaporated. The residue was treated with aqueous NaHCO_3_ (50 mL) and extracted with ethyl acetate (3 × 150 mL). The organic layer was dried (MgSO_4_), filtered, and evaporated to give crude bromide **8**. A suspension of K_2_CO_3_ (27.68 mmol), 18-crown-6 (10.36 mmol), and 6-chloropurine (13.81 mmol) or 2-amino-6-chloropurine (13.81 mmol) in *N,N*-dimethylformamide (100 mL) was stirred under N_2_ at 85 °C for 3 h. To this mixture, a solution of **8** in *N*,*N*-dimethylformamide (DMF) (10 mL) was added and the reaction mixture was stirred at 55 °C for 7 h, filtered, and evaporated. The residue was purified by silica gel column chromatography (hexanes:ethyl acetate = 1:1) to give the base condensed product **9**, **10**, **18**, or **19**.

*9-((2-O-tert-Butyldiphenylsilyloxyethylselanylmethyl)-6-chloro-9H-purin* (**9**). Yield: 46%; colorless syrup; UV (MeOH) λ_max_ 264 nm; ^1^H-NMR (400 MHz, CDCl_3_) δ 8.71 (s, 1H), 8.17 (s, 2H), 7.68–7.65 (m, 4H), 7.46–7.37 (m, 6H), 5.43 (s, 2H), 3.93 (t, *J* = 6 Hz, 2H), 2.88 (t, *J* = 6.4 Hz, 2H), 1.05 (s, 9H); ^13^C-NMR (100 MHz, CDCl_3_) δ 152.13, 151.51, 151.24, 144.86, 135.54, 133.11, 131.69, 129.89, 127.79, 64.07, 34.20, 27.93, 26.81, 19.17; MS (ESI) *m*/*z* 531.0888 (M + H)^+^; Anal. Calcd. for C_24_H_27_ClN_4_OSeSi: C, 54.39; H, 5.14; N, 10.57. Found: C, 54.89; H, 4.99; N, 10.43.

*9-((2-O-tert-Butyldiphenylsilyloxyethylselanyl)methyl)-6-chloro-9H-purin-2-amine* (**10**). Yield: 45%; white solid; m.p. 115–120 °C; UV (MeOH) λ_max_ 310 nm; ^1^H-NMR (400 MHz, CDCl_3_) δ 7.81 (s, 1H), 7.6–7.65 (m, 4H), 7.46–7.37 (m, 6H), 5.22 (s, 2H), 3.92 (t, *J* = 6.8 Hz, 2H), 2.86 (t, *J* = 6.4 Hz, 2H), 1.05 (s, 9H); MS (ESI) *m*/*z* 546.0995 (M + H)^+^; Anal. Calcd. for C_24_H_28_ClN_5_OSeSi: C, 52.89; H, 5.18; N, 12.85. Found: C, 52.99; H, 4.98; N, 12.45.

*9-((2-Phenyl-1, 3-dioxan-5-ylselanyl)methyl)-6-chloro-9H-purin* (**18**). Yield: 45%; colorless syrup; UV (MeOH) λ_max_ 264 nm; ^1^H-NMR (400 MHz, CDCl_3_) δ 8.79 (s, 1H), 8.27 (s, 1H), 7.41–7.39 (m, 2H), 7.33–7.31 (m, 3H), 5.46 (s, 2H), 5.43 (s, 1H), 4.29 (dd, *J* = 4.6 Hz, 11.4 Hz, 2H), 3.78 (t, *J* = 11.8 Hz, 2H), 3.65–3.57 (m, 1H); MS (ESI) *m*/*z* 411.0113 (M + H)^+^; Anal. Calcd. for C_16_H_15_ClN_4_O_2_Se: C, 46.90; H, 3.69; N, 13.67. Found: C, 46.56; H, 3.39; N, 13.89.

*9-((2-Phenyl-1,3-dioxan-5-ylselanyl)methyl)-6-chloro-9H-purin-2-amine* (**19**). Yield: 45%; white solid; m.p. 164–166 °C; UV (MeOH) λ_max_ 310 nm; ^1^H-NMR (400 MHz, CDCl_3_) δ 7.88 (s, 1H), 7.43–7.40 (m, 2H), 7.36–7.32 (m, 3H), 5.44 (s, 1H), 5.27 (s, 2H), 5.24 (s, 2H), 4.32 (dd, *J* = 4.5 and 11.4 Hz, 2H), 3.82–3.76 (m, 2H), 3.69–3.63 (s, 1H); ^13^C-NMR (125 MHz, CD_3_OD) δ 159.3, 153.4, 151.8, 141.4, 137.6, 129.1, 128.3, 125.9, 125.2, 101.4, 71.4. 35.4, 33.4; MS (FAB) *m*/*z* 426.0117 (M + H)^+^; Anal. Calcd. for C_16_H_16_ClN_5_O_2_Se: C, 45.24; H, 3.80; N, 16.49. Found: C, 45.33; H, 4.10; N, 16.55.

### 3.2. General Procedure for TBDPS Removals

To a solution of **9** or **10** (4.036 mmol) in tetrahydrofuran (THF) (40 mL), tetra-*n*-butylammonium fluoride (2.06 mmol, 1 M solution in THF) was added under N_2_, and the reaction mixture stirred at room temperature for 1 h and evaporated. The residue was purified by column chromatography (hexanes:ethyl acetate = 1:9) to give **11** or **12**, respectively.

*2-((6-Chloro-9H-purin-9-yl)methylselanyl) ethanol* (**11**). Yield: 92%; white solid; m.p. 98–100 °C; UV (MeOH) λ_max_ 264 nm; ^1^H-NMR (400 MHz, CDCl_3_) δ 8.79 (s, 1H), 8.32 (s, 1H), 5.61 (s, 2H), 4.02 (q, *J* = 5.6 Hz, 2H), 2.88 (t, *J* = 5.6 Hz, 2H), 2.65 (t, *J* = 5.6 Hz, 1H); ^13^C-NMR (100 MHz, CDCl_3_) δ 152.30, 151.72, 151.69, 145.47, 132.03, 63.41, 34.54, 28.28; MS (ESI) *m*/*z* 290.9713 (M + H)^+^.

*2-((2-Amino-6-chloro-9H-purin-9-yl)methylselanyl) ethanol* (**12**). Yield: 45%; white solid; m.p. 150–152 °C; UV (MeOH) λ_max_ 310 nm; ^1^H-NMR (400 MHz, DMSO-*d*_6_) δ 8.22 (s, 1H), 6.95 (br s, 2H, exchangeable), 5.35 (s, 2H), 3.59 (t, *J* = 6.4 Hz, 2H), 2.83 (t, *J* = 6.8 Hz, 2H); ^13^C-NMR (100 MHz, DMSO-*d*_6_) δ 159.8, 153.6, 149.5, 142.7, 123.4, 61.4, 33.8, 27.7; MS (ESI) *m*/*z* 307.9807 (M + H)^+^.

### 3.3. General Procedure for the 1,3-Benzylidene Removals

To a solution of **18** or **19** (7.06 mmol) in MeOH (20 mL), iodine (0.2 mL, 0.1 M solution in MeOH) was added and the reaction mixture was heated at 60 °C for 4 h. Then, the reaction mixture was quenched with few drops of aqueous sodium thiosulfate and evaporated. The residue was purified by silica gel column chromatography (CH_2_Cl_2_:MeOH = 24:1) to give **20** or **21**, respectively.

*2-((6-Chloro-9H-purin-9-yl)methylselanyl) propane-1, 3-diol* (**20**). Yield: 88%; white solid; m.p. 140–143 °C; UV (MeOH) λ_max_ 263 nm; ^1^H-NMR (400 MHz, CD_3_OD) δ 8.75 (s, 1H), 8.69 (s, 1H), 5.68 (s, 2H), 3.79 (s, 2H), 3.77 (s, 2H), 3.27 (s, 1H); ^13^C-NMR (100 MHz, CD_3_OD) δ 153.89, 153.78, 152.11, 148.97, 133.23, 64.54, 49.63, 35.80; MS (FAB) *m*/*z* 326.1516 (M + H)^+^.

*2-((2-Amino-6-chloro-9H-purin-9-yl)methylselanyl) propane-1,3-diol* (**21**). Yield: 88%; white solid; m.p. 160–162 °C; UV (MeOH) λ_max_ 310 nm; ^1^H-NMR (400 MHz, CD_3_OD) δ 8.18 (s, 1H), 5.45 (s, 2H), 3.85–3.76 (m, 4H), 3.3–3.33 (m, 1H); ^13^C-NMR (100 MHz, CD_3_OD + CDCl_3_) δ 162.1, 155.5, 152.3, 144.7, 125.6, 71.8, 64.4, 48.8, 35.0; MS (ESI) *m*/*z* 337.9915 (M + H)^+^; Anal. Calcd. for C_9_H_12_ClN_5_O_2_Se: C, 32.11; H, 3.59; N, 20.80. Found: C, 32.01; H, 3.91; N, 20.56.

### 3.4. Conversion of 6-Chloro Derivatives to 6-Keto Derivatives

A solution of 6-chloro derivative **11**, **12**, **20**, or **21** (0.358 mmol), 2-mercaptoethanol (1.297 mmol) and NaOMe (1.797 mmol) in methanol (15 mL) was refluxed for 4 days at 75 °C. After completion of the reaction, the reaction mixture was cooled and neutralized with acetic acid. The solvent was removed under reduced pressure and the residue was chromatographed (CH_2_Cl_2_:MeOH = 7:1) to give the 6-keto derivative **3a**, **4a**, **3d**, or **4d**.

*9-((2-Hydroxyethylselanyl)methyl)-1H-purin-6-(9H)-one* (**3a**). Yield: 80%; white solid; m.p. 225–227 °C; UV (MeOH) λ_max_, 248 nm; ^1^H-NMR (400 MHz, DMSO-*d*_6_) δ 8.16 (s, 1H), 8.06 (s, 1H), 5.44 (s, 2H), 4.89 (br s, 1H, exchangeable), 3.58–3.56 (m, 2H), 2.81 (t, *J* = 6.8 Hz, 2H); ^13^C-NMR (100 MHz, DMSO-*d*_6_) δ 156.64, 148.03, 145.87, 139.96, 124.13, 61.29, 33.95, 27.55; MS (ESI) *m*/*z* 275.0041 (M + H)^+^; Anal. Calcd. for C_8_H_10_N_4_O_2_Se: C, 35.18; H, 3.69; N, 20.51. Found: C, 35.19; H, 3.56; N, 20.11.

*9-((2-Hydroxyethylselanyl)methyl)-2-amino-1H purin-6(9H)-one* (**4a**) [12]. Yield: 94%; white solid: m.p. 266–268 °C; UV (MeOH) λ_max_ 259 nm; ^1^H-NMR (400 MHz, DMSO-*d*_6_) δ 10.60 (br s, 1H, exchangeable with D_2_O), 7.78 (s, 1H), 6.49 (br s, 2H), 5.23 (s, 2H), 4.86 (t, *J* = 5.3 Hz, 1H), 3.58 (q, *J* = 6.5 Hz, 2H), 2.79 (t, *J* = 6.8 Hz, 2H); ^13^C-NMR (100 MHz, DMSO-*d*_6_) δ 156.7, 153.7, 150.9, 137.1, 116.6, 61.4, 33.4, 27.3; MS (ESI) *m*/*z* 290.0152 (M + H)^+^; Anal. Calcd. for C_8_H_11_N_5_O_2_Se: C, 33.34; H, 3.85; N, 24.30. Found: C, 33.14; H, 4.15; N, 24.01.

*9-((1,3-Dihydroxypropan-2-ylselanyl)methyl)-1H purin-6(9H)-one* (**3d**). Yield: 85%; white solid; m.p. 189–182 °C; UV (MeOH) λ_max_ 247 nm; ^1^H-NMR (400 MHz, DMSO-*d*_6_) δ 8.17 (s, 1H), 8.06 (s, 1H), 5.46 (s, 2H), 4.84 (br s, 2H, exchangeable), 3.65–3.56 (m, 4H), 3.21–3.17 (m, 1H); ^13^C-NMR (100 MHz, DMSO-*d*_6_) δ 156.68, 148.01, 145.84, 139.93, 124.06, 61.49, 47.30, 33.23; MS (ESI) *m*/*z* 305.0142 (M + H)^+^; Anal. Calcd. for C_9_H_12_N_4_O_3_Se: C, 35.66; H, 3.99; N, 18.48. Found: C, 35.26; H, 4.13; N, 18.08.

*9-((1,3-Dihydroxypropan-2-ylselanyl)methyl)-2-amino-1H-purin-6(9H)-one* (**4d**) [12]. Yield: 70%; white solid; m.p. 204–207 °C; UV (MeOH) λ_max_ 258 nm; ^1^H-NMR (400 MHz, DMSO-*d*_6_) δ 10.69 (br s, 1H, exchangeable with D_2_O), 7.78 (s, 1H), 6.59 (s, 2H, exchangeable with D_2_O), 5.5 (s, 2H), 4.84 (t, *J* = 5.3 Hz, 2H, exchangeable with D_2_O), 3.69–3.64 (m, 2H), 3.62-3.56 (m, 2H, exchangeable with D_2_O), 3.21–3.16 (m, 1H); ^13^C-NMR (100 MHz, DMSO-*d*_6_) δ 156.8, 153.8, 150.9, 137.0, 116.6, 61.6, 47.0, 32.8; MS (ESI) *m*/*z* 320.0254 (M + H)^+^; Anal. Calcd. for C_9_H_13_N_5_O_3_Se: C, 33.97; H, 4.12; N, 22.01. Found: C, 34.12; H, 4.42; N, 22.37.

### 3.5. Conversion of 6-Chloro Derivatives to N^6^-Methylamino Derivatives

A solution of 6-chloro derivative **11**, **12**, **20,** or **21** (0.342 mmol) and methylamine (10 mL, 40% aqueous solution) in methanol (10 mL) was heated at 85 °C for 48 h in a steel bomb. After completion of the reaction, the solvent was removed and the residue was purified by silica gel column chromatography (CH_2_Cl_2_:MeOH = 20:1) to give the *N*^6^-methylamino derivative **3b**, **4b**, **3e**, or **4e**.

*2-((6-(Methylamino)-9H-purin-9-yl)methylselanyl) ethanol* (**3b**). Yield: 70%; white solid; m.p. 171–173 °C; UV (MeOH) λ_max_ 266 nm; ^1^H-NMR (400 MHz, CD_3_OD) δ 8.26 (s, 1H), 8.17 (s, 1H), 5.49 (s, 2H), 3.73 (t, *J* = 6.6 Hz, 2H), 3.10 (broad s, 3H), 2.84 (t, *J* = 6.6 Hz, 2H); ^13^C-NMR (100 MHz, CD_3_OD) δ 157.58, 154.69, 150.18, 142.64, 121.46, 64.11, 35.66, 28.88; MS (ESI) *m*/*z* 288.0363 (M + H)^+^; Anal. Calcd. for C_9_H_13_N_5_OSe: C, 37.77; H, 4.58; N, 24.47. Found: C, 37.78; H, 4.55; N, 24.31.

*2-((2-Amino-6-(methylamino)-9H-purin-9-yl)methylselanyl) ethanol* (**4b**). Yield: 85%; white solid; m.p. 183–185 °C; UV (MeOH) λ_max_ 282 nm; ^1^H-NMR (400 MHz, CD_3_OD) δ 7.81 (s, 1H), 5.35 (s, 2H), 3.75 (t, *J* = 6.7 Hz, 2H), 3.04 (broad s, 3H), 2.85 (t, *J* = 6.6 Hz, 2H); ^13^C-NMR (75 MHz, CD_3_OD) δ 162.91, 158.07, 139.54, 115.59, 64.28, 64.28, 35.28, 31.54, 28.58; MS (ESI) *m*/*z* 303.0472 (M + H)^+^; Anal. Calcd. for C_9_H_14_N_6_OSe: C, 35.89; H, 4.69; N, 27.90. Found: C, 36.18; H, 4.32; N, 28.01.

*2-((6-(Methylamino)-9H-purin-9-yl)methylselanyl)propane-1,3-diol* (**3e**). Yield: 50%; white solid; m.p. 180–183 °C; UV (MeOH) λ_max_ 266 nm; ^1^H-NMR (400 MHz, DMSO-*d*_6_) δ 8.24 (s, 1H), 8.22 (s, 1H), 7.69 (br s, 1H, exchangeable), 5.48 (s, 2H), 4.84 (t, *J* = 5.1 Hz, 2H, exchangeable), 3.67–3.56 (m, 4H), 3.25–3.21 (m, 1H), 2.96 (br s, 3H); ^13^C-NMR (75 MHz, DMSO-*d*_6_) δ 154.90, 152.56, 148.14, 140.19, 119.20, 61.55, 47.26, 33.03, 27.01; MS (ESI) *m*/*z* 318.0469 (M + H)^+^; Anal. Calcd. for C_10_H_15_N_5_O_2_Se: C, 37.98; H, 4.78; N, 22.15. Found: C, 37.99; H, 4.36; N, 22.01.

*2-((2-Amino-6-(methylamio)-9H-purin-9-yl)methylselanyl) propane-1,3-diol* (**4e**). Yield: 50%; white solid; m.p. 140–143 °C; UV (MeOH) λ_max_ 282 nm; ^1^H-NMR (400 MHz, DMSO-*d*_6_) δ 7.77 (s, 1H), 7.16 (br s, 1H, exchangeable), 5.88 (s, 2H, exchangeable), 5.28 (s, 2H), 4.82 (t, *J* = 5.2 Hz, 2H, exchangeable), 3.70–3.65 (m, 2H), 3.63-3.57 (m, 2H), 3.21–3.16 (m, 1H), 2.89 (br s, 3H); ^13^C-NMR (100 MHz, DMSO-*d*_6_) δ 160.28, 155.38, 136.57, 113.43, 105.24, 61.67, 46.88, 32.61, 26.90, 26.85; MS (ESI) *m*/*z* 333.0573 (M + H)^+^; Anal. Calcd. for C_10_H_16_N_6_O_2_Se: C, 36.26; H, 4.87; N, 25.37. Found: C, 36.56; H, 443; N, 25.08.

### 3.6. Conversion of 6-Chloro Derivatives to N^6^-Amino Derivatives

6-Chloro derivative **11**, **12**, **20**, or **21** (0.342 mmol) and NH_3_/t-butanol (10 mL) were taken in a steel bomb and heated at 85 °C for 12 h. The solvent was removed and the residue was purified by silica gel column chromatography (CH_2_Cl_2_:MeOH = 24:1) to give *N*^6^-amino derivative **3c**, **4c**, **3f**, **4f**, or **4g**.

*2-((6-Amino-9H-purin-9-yl)methylselanyl)ethanol* (**3c**). Yield: 95%; white solid; m.p. 175–177 °C; UV (MeOH) λ_max_ 260 nm; ^1^H-NMR (400 MHz, CD_3_OD) δ 8.25 (s, 1H), 8.23 (s, 1H), 5.52 (s, 2H), 3.74 (t, *J* = 6.4 Hz, 2H), 2.86 (t, *J* = 6.4 Hz, 2H); ^13^C-NMR (100 MHz, CD_3_OD) δ 157.41, 153.94, 150.52, 142.52, 120.17, 63.35, 34.95, 28.16; MS (ESI) *m*/*z* 274.0202 (M + H)^+^; Anal. Calcd. for C_8_H_11_N_5_OSe: C, 35.30; H, 4.07; N, 25.73. Found: C, 35.20; H, 4.47; N, 25.43.

*2-((2,6-Diamino-9H-purin-9-yl)methylselanyl) ethanol* (**4c**). Yield: 70%; white solid; m.p. 84–86 °C; UV (MeOH) λ_max_ 281 nm; ^1^H-NMR (400 MHz, DMSO-*d*_6_) δ 7.80 (s, 1H), 6.73 (s, 2H, exchangeable), 5.86 (s, 2H, exchangeable), 5.28 (s, 2H), 4.89 (t, *J* = 5.3 Hz, 1H, exchangeable), 3.60 (q, *J* = 6.3 Hz, 2H), 2.81 (t, *J* = 6.7 Hz, 2H); ^13^C-NMR (75 MHz, DMSO-*d*_6_) δ 160.32, 156.07, 151.42, 136.99, 113.11, 61.46, 33.28, 27.22; MS (ESI) *m*/*z* 289.0314 (M + H)^+^; Anal. Calcd. for C_8_H_12_N_6_OSe: C, 33.46; H, 4.21; N, 29.26. Found: C, 33.20; H, 4.41; N, 29.43.

*2-((6-Amino-9H-purin-9-yl)methylselanyl)propane-1,3-diol* (**3f**). Yield: 80%; white solid; m.p. 186–188 °C; UV (MeOH) λ_max_ 260 nm; ^1^H-NMR (400 MHz, DMSO-*d*_6_) δ 8.23 (s, 1H), 8.16 (s, 1H), 7.23 (s, 2H, exchangeable), 5.47 (s, 2H), 4.85 (t, *J* = 5.1 Hz, 2H, exchangeable), 3.67–3.57 (m, 4H), 3.24–3.21 (m, 1H); ^13^C-NMR (100 MHz, DMSO-*d*_6_) δ 155.93, 152.53, 149.11, 140.47, 118.72, 61.57, 47.24, 32.96; MS (ESI) *m*/*z* 304.0292 (M + H)^+^; Anal. Calcd. for C_9_H_13_N_5_O_2_Se: C, 35.77; H, 4.34; N, 23.18. Found: C, 35.89; H, 4.12; N, 23.02.

*2-((2,6-Diamino-9H-purin-9-yl)methylselanyl)propane-1,3-diol* (**4f**). Yield: 75%; white solid; m.p. 193–196 °C; UV (MeOH) λ_max_ 282 nm; ^1^H-NMR (400 MHz, DMSO-*d*_6_) δ 7.79 (s, 1H), 6.67 (s, 2H, exchangeable), 5.81 (d, *J* = 2.8 Hz, 2H, exchangeable), 5.28 (s, 2H), 4.84 (t, *J* = 5.1 Hz, 1H, exchangeable), 3.71–3.66 (m, 2H), 3.63–3.57 (m, 2H), 3.29 (s, 1H, exchangeable), 3.23–3.16 (m, 1H); ^13^C-NMR (100 MHz, DMSO-*d*_6_) δ 160.20, 156.03, 151.36, 136.98, 113.09, 61.66, 61.55, 46.84, 32.61; MS (ESI) *m*/*z* 319.0414 (M + H)^+^; Anal. Calcd. for C_9_H_14_N_6_O_2_Se: C, 34.08; H, 4.45; N, 26.49. Found: C, 34.48; H, 4.17; N, 26.83.

*2-((2-Amino-6-(cyclopropylamino)-9H-purin-9-yl)methylselanyl)propane-1,3-diol* (**4g**). To a solution of **44** (100 mg, 0.296 mmol) in ethanol (10 mL) in a steel bomb, cyclopropylamine (0.103 mL, 1.486 mmol) and triethylamine (0.272 mL, 1.950 mmol) were added and the mixture was heated at 100 °C for 48 h. After completion of the reaction, the solvent was removed and the residue was purified by silica gel chromatography (CH_2_Cl_2_:MeOH = 20:1 to give **4d** (52 mg, 55%) as a white solid: m.p. 140–143 °C; UV (MeOH) λ_max_ 285 nm; ^1^H-NMR (400 MHz, DMSO-*d*_6_) δ 7.79 (s, 1H), 7.29 (br s, 1H, exchangeable), 5.88 (s, 2H, exchangeable), 5.29 (s, 2H), 4.84 (t, *J* = 5.2 Hz, 2H, exchangeable), 3.71–3.66 (m, 2H), 3.63–3.57 (m, 2H), 3.22–3.19 (m, 1H), 3.03 (br s, 1H), 0.68-0.62 (m, 2H), 0.59–0.58 (m, 2H); ^13^C-NMR (100 MHz, DMSO-*d*_6_) δ 160.19, 155.81, 150.94, 136.73, 113.35, 69.68, 61.63, 46.91, 32.66, 23.86, 6.44; MS (ESI) *m*/*z* 359.0731 (M + H)^+^; Anal. Calcd. for C_12_H_18_N_6_O_2_Se: C, 40.34; H, 5.08; N, 23.52. Found: C, 40.14; H, 5.17; N, 23.12.

### 3.7. Antiviral Activity and Cytotoxicity Assays

Antiviral activity was measured using a standard cytopathic (CPE) inhibition assay as described before [13]. Briefly, Vero cells in stationary phase were infected with the virus at a multiplicity of infection of 2–4 CCID50 (50% cell culture inhibitory dose) per each well of 96-well plates. After 2 h of adsorption at 37 °C, the liquid was aspirated and 100 μL of Dulbeco’s modified eagle’s media (DMEM)/2% fetal bovine serum (FBS) containing a compound was applied to each well in duplicate for each concentration and further incubated for 3 days. Antiviral activity was measured by MTT assay and expressed as the EC_50_. Cytocidal assay was performed as a control experiment for the antiviral assay. It was carried out simultaneously with the antiviral assay described above using mock instead of virus for infection, and cell viability was measured by MTT assay. The concentration of the compound responsible for 50% reduction of cell growth was calculated and expressed as CC_50_.

## 4. Conclusions

We have synthesized various acyclic seleno-purine nucleosides **3a**–**f** and **4a**–**g**, and evaluated them for anti-herpetic activity. The key diselenides **6** and **16** were synthesized by treating bromide **5** and mesylate **15** with selenium powder and hydrazine hydrate in aqueous KOH solution, respectively. The glycosyl donors **8** and **17** were synthesized by treating diselenides **7** and **16** with NaBH_4_ followed by trapping with methylene bromide, and then condensed with 6-chloropurine or 2-amino-6-chloropurine anion in a S_N_2 manner. Among the compounds tested, seleno-acyclovir (**4a**) showed the most potent anti-HSV-1 and HSV-2 activities, while the seleno-ganciclovir analogue **4e** exhibited the most potent anti-HCMV activity. It seems that 2,6-diaminopurine nucleosides might be converted to seleno-acyclovir (**4a**) and seleno-ganciclovir (**4d**) by cellular nucleoside deaminases, serving as prodrugs of **4a** and **4d**. Although we could not discover more potent compounds than the reference compounds, acyclovir (**1**) or ganciclovir (**2**), it is expected that these synthesized nucleosides can be a new template for the design of novel acyclic nucleoside analogues.

## Figures and Tables

**Table 1:** Antiviral activities of all the final nucleosides **3a**-**f** and **4a**-**g**.

Compound (X, R_1_, R_2_)	EC_50_ (µM) ^a^				CC_50_ (µM) ^b^
	HSV-1	HSV-2	VZV	HCMV	HEL299
**3a** (X = H, R_1_ = OH, R_2_ = H)	>100	>100	>100	>100	>100
**3b** (X = H, R_1_ = NHMe, R_2_ = H)	>100	>100	>100	>100	>100
**3c** (X = H, R_1_ = NH_2_, R_2_ = H)	>100	>100	>100	>100	>100
**3d** (X = CH_2_OH, R_1_ = OH, R_2_ = H)	>100	>100	>100	>100	>100
**3e** (X = CH_2_OH, R_1_ = NHMe, R_2_ = H)	>100	>100	>100	>100	>100
**3f** (X = CH_2_OH, R_1_ = NH_2_, R_2_ = H)	>100	>100	>100	65.2	>100
**4a** (X = H, R_1_ = OH, R_2_ = NH_2_) ^c^	1.47	6.34	>100	>100	>100
**4b** (X = H, R_1_ = NHMe, R_2_ = NH_2_)	14.3	17.6	>100	>100	>100
**4c** (X = H, R_1_ = NH_2_, R_2_ = NH_2_)	15.4	23.2	>100	>100	>100
**4d** (X = CH_2_OH, R_1_ = OH, R_2_ = NH_2_) ^c^	>100	>100	>100	53.1	>100
**4e** (X = CH_2_OH, R_1_ = NHMe, R_2_ = NH_2_)	>100	>100	>100	32.1	>100
**4f** (X = CH_2_OH, R_1_ = NH_2_, R_2_ = NH_2_)	>100	>100	>100	34.3	>100
**4g** (X = CH_2_OH, R_1_ = NH-cyclopropyl, R_2_ = NH_2_)	>100	>100	>100	41.1	>100
**1** (Acyclovir)	0.66	1.02	6.4	18.9	>100
**2** (Ganciclovir)	0.90	1.40	11.1	2.14	>100

^a^ The effective concentration required to inhibit virus-induced cytopathic effect by 50%; ^b^ The cytotoxic concentration of the compound responsible for 50% reduction of cell viability; ^c^ Reference [11].
